# Maintenance of Pest-House Close to School

**Published:** 1900-10

**Authors:** 


### Abstracts and Selections.

### Maintenance of Pest-House Close to School.

The court of civil appeals of Texas has approved, in the case of Thompson vs. Kimbrough, of the suppression by injunction of the maintenance of a pest-house for small-pox suspects and those afflicted with the disease within 192 feet of a public school, on property purchased by the county. One of the contentions was that the school district trustees had no legal right to maintain a suit for injunction against the county judge, commissioners court and county health officer. But the court answers that, if the ground of interest alone be considered, it would certainly appear that the trustees had such interest in the premises as would give them standing as complainants against persons attempting to injure the school property and interfere with the operation of the school. Then it was contended that the law invested the commissioners court, in connection with the county health officer, with exclusive and final jurisdiction in the matter of establishing and maintaining pest-houses and detention camps to prevent the spread of small-pox and other infectious and contagious diseases, and that the exercise of this jurisdiction was not subject to review by any judicial tribunal. Referring to the Texas statutes on the subject of quarantine, the court says that they authorize cities and counties to establish and maintain quarantine when thought necessary for the preservation of the public health, and require their co-operation with the State authorities in the maintenance of a quarantine proclaimed by the governor of the State. It is also required that the county judge of each county shall appoint, after each general election, a "county physician," who is made the health officer of the county. He is charged with the duty of establishing, maintaining and enforcing local quarantine, when declared by proclamation of the commissioners court. The commissioners court is authorized, whenever it is believed that they are threatened with the introduction or dissemination of a dangerous, contagious or infectious disease that may be guarded against by quarantine, to establish and maintain detention stations or camps; to provide hospitals, tents or pest-houses for the sick, etc., and the county is required to meet the expenses. Now, it may, and perhaps should be, admitted, the court goes on to say, that the exercise of the authority of the commissioners court to proclaim quarantine, and to have it established, maintained and enforced through the county physician, is not subject to review by judicial tribunals. But, the court says, it does not necessarily follow that all of the acts of the officers and agents of the county in relation to the subject matter are beyond the reach of judicial authority. The authority of the commissioners court to proclaim and cause the enforcement of quarantine does not carry with it the arbitrary power in the officers and agents of the county to unnecessarily violate the rights of others. The existence of such authority does not imply that the officers and agents of the county may establish and maintain a nuisance, either public or private, not necessary to the end to be accomplished. The establishment of a detention station and pest-house in which to place, respectively, persons who have been exposed to small-pox, and those afflicted with the disease, in such close proximity to a public school that the germs of the disease may and probably would be disseminated among the children attending such school, the court holds, furthermore, would be the worst character of nuisance, and would violate the rights of those interested in the school. To uphold such an act, it adds, would not strengthen the county's efforts to prevent the spread of the loathsome and dangerous disease, but it would support the very means of its dissemination, and thereby defeat the humane object which the law designs to be accomplished.—*Journal of the American Medical Association.*

